# Ultrasound assessments of organs and blood vessels before and after 40 days isolation in a cavern (deep time experiment 2021)

**DOI:** 10.3389/fphys.2023.1174565

**Published:** 2023-04-24

**Authors:** Philippe Arbeille, Kathryn A. Zuj, Stephane Besnard, Benoit Mauvieux, Corentin Hingrand, Pierre-Louis Delaunay, Richard L. Hughson, Christian Clot

**Affiliations:** ^1^ UMPS-CERCOM (Unit Med Physiol Spatiale) Faculte de Medicine Universite de Tours, Tours, France; ^2^ Department Explorations Neurologiques et ORL, CHU, Caen, France; ^3^ COMETE U1075, Unicaen—Inserm, Gip Cyceron, Caen, France; ^4^ Schlegel-University of Waterloo Research Institute for Aging, Waterloo, ON, Canada; ^5^ Department Adaptation Comportementale et Fonctionnel Aux Changements Human Adaptation Institute, Paris, France

**Keywords:** echography, organ vessel imaging, isolation, spaceflight, cavern, echography

## Abstract

**Introduction:** Spaceflight simulation studies like confinement in small volume habitat with limited physical activity have reported even after 60 days an abnormal arterial wall adaptation with increase thickness or stiffness. The purpose of the current study was to determine the effects on blood vessel and organ structure of 40 days of isolation in a huge habitat with intensive physical activity.

**Method:** Data were collected from 14 individuals (7 male) who isolated in a cavern for 40-days while performing normal daily activities without time references. Ultrasound assessments were performed pre- and post-isolation using a teleoperated system with eight different acoustic windows to obtain 19 measurements on 12 different organ/vascular structures which included the common carotid artery, femoral artery, tibial artery, jugular vein, portal vein, bile duct, kidney, pancreas, abdominal aorta, cervical and lumbar vertebral distance, and Achilles tendon.

**Results:** Common carotid artery measures, including the intima media thickness, stiffness index, and the index of reflectivity measured from the radiofrequency signal, were not changed with isolation. Similarly, no differences were found for femoral artery measurements or measurements of any of the other organs/vessels assessed. There were no sex differences for any of the assessments.

**Discussion:** Results from this study indicate a lack of physiological effects of 40-days of isolation in a cavern, contrary to what observed in previous 60 days confinement. This suggests a potential protective effect of sustained physical activity, or reduced environmental stress inside the huge volume of the confined facility.

## Introduction

Exposure to microgravity results in multiple physiological adaptations. After 6-months on the International Space Station (ISS), common carotid arterial wall thickness increases, and wall distensibility reduces (Hughson et al., 2016; Arbeille et al., 2017). The ultrasound index of reflectivity of the carotid arterial wall, measured from the radiofrequency signal, also increase suggesting changes in wall composition (Arbeille et al., 2021a) potentially related to glucose or calcium metabolism (Hughson et al., 2016). Intracranial venous blood velocity increase with spaceflight (Arbeille et al., 2021b) associated with altered cerebral structure compressing intracranial veins (Roberts et al., 2017). Spaceflight also increases jugular vein volume (Arbeille et al., 2015; Arbeille et al., 2021b) and alters jugular vein blood flow leading to an increased risk of thrombus formation (Marshall-Goebel et al., 2019). While it is believed that these adaptations primarily result from the fluid shifts associated with microgravity exposure, other factors such as physical and psychological stress from induced confinement or a lack of physical activity may also contribute to the observed adaptations.

The Mars 500 study confined six male participants in a relatively small (550 m^3^) isolation module for 520 days to simulate the conditions associated with a mission to Mars. Interestingly, although the participant population was small (6 subjects) and they were not exposed to real or simulated microgravity, arterial wall thickness increased with this change occurring after only 60 days of confinement (Arbeille et al., 2014a). Similar results were found after 60 days of confinement during the 6 months CELSS study with 4 subjects (Yuan et al., 2019). For both studies, participants resided in relatively small habitats with reduced daily physical activity, similar to what is experience by astronauts during spaceflight. Therefore, as these studies showed similar physiological responses to spaceflight but in normal gravity, reduced physical activity and physical and mental stress from confinement are supposed to be major contributing factors to the responses.

The current study was designed to investigate if 40 days of isolation in a cavern while smaller compare to previous confinement studies had an effect on various vascular and organ structures. Contrary to the previous confinement studies previously mentioned, this study required participants to conduct normal daily sustained activities in a relatively large confinement space with no external clues regarding time of day. Nevertheless, it was hypothesized that such isolation would results in changes in vessel and organ structure after 40 days similar to those observed during the Mars 500 and CELSS studies after 60 days and with spaceflight after 30 days.

## Research design and methods

### Subjects

Data were collected from 14 individuals who participated in the Deep Time experiment (7 male; 34 ± 7 years of age; 176 ± 6 cm height; 74 ± 7 kg body mass). All study protocols and procedures were in accordance with the Declaration of Helsinki and were approved by the local research ethics committee (CPP Normandy ID-RCB 2021-A0474-7). Each participant gave written informed consent before participating in the study.

### Experimental protocol

For the study, participants resided in a cavern for 40 days with no time reference during this period. The cavern had a volume of several thousand cubic metres, ceiling height of 10 m, average temperature of 10°C, and 98% humidity. Sleeping quarters were located approximately 800 m from the living space requiring participants to walk this distance several times each day. During the isolation period, participants also performed missions to walk for water (4–5 times, ∼2 h each time) and for exploration (4 times at ∼8 h each time). Finally, participants also travelled to the cavern entrance four times during the isolation period to dispose of waste (∼40 min walk). The average daily caloric intake was 2,600 kcal, but was not strictly controlled.

### Measurements

Ultrasound assessments were performed during the 1-week ambulatory period 7 and 4 days before participants entered the cavern and again 5 and 20 h after they emerged after 40 days of isolation. All ultrasound assessments were done with the participant in a relaxed seated position with the chair backrest inclined 40° from the vertical. Images were collected using a teleoperated ultrasound system as a simulation of space exploration aboard the ISS. This method, previously validated in a clinical setting (Arbeille et al., 2016) and with astronauts on the ISS (Arbeille et al., 2018)^,^ had the additional benefit of limiting contact with participants to reduce the risks associated with the global pandemic occurring during this study.

**FIGURE 1:**
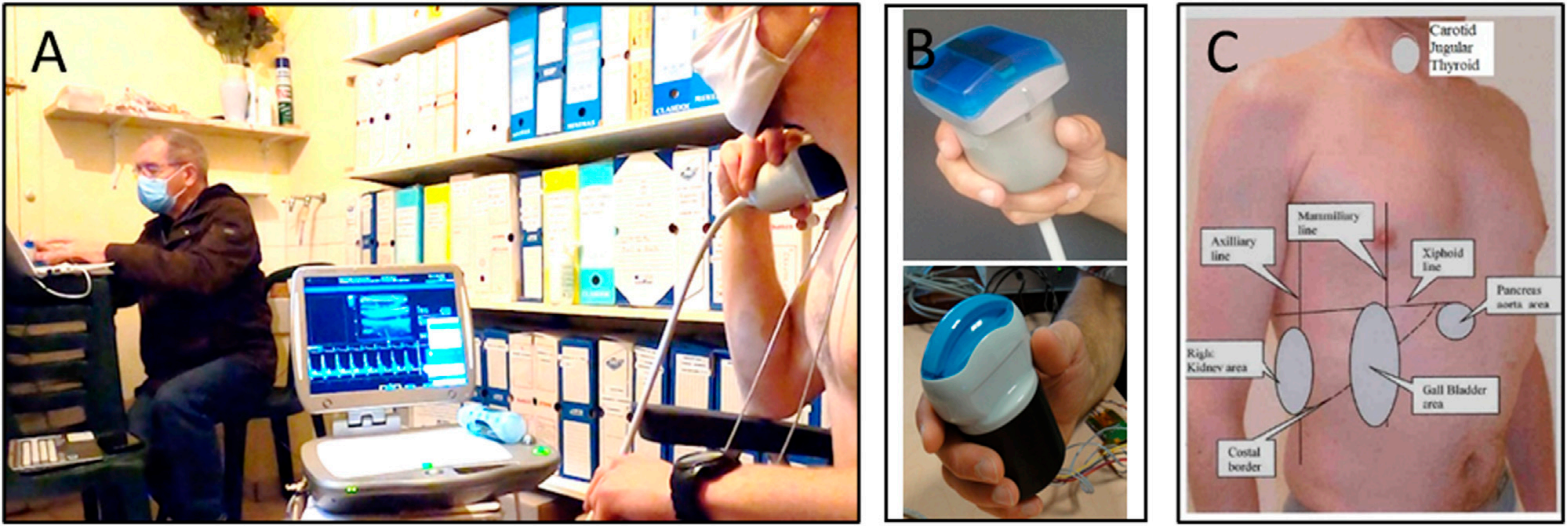
Images show the experimental setup for the ultrasound data collection. In panel **(A)** the participant is holding the motorized probe in place (right) while the expert sonographer (left) teleoperates the system to acquire images for analysis. Panel **(B)** depicts the 3.5 MHz and 17 MHz motorized probes used for the investigation. Finally, panel **(C)** shows the map of the various acoustic windows of each organ (ex., gall bladder, kidney) and landmarks (ex., mamillary, axillary line) used for the ultrasound assessments that was provided to the participant as reference for proper probe placement.

This teleoperated ultrasound system was similar to the one currently in use on the ISS. It consisted of a commercially available ultrasound machine (Orcheolite TE, Sonoscanner, Paris, France) that was modified for teleoperation using motorized probes (Arbeille et al., 2016; Arbeille et al., 2018)^.^ For the assessment, participants positioned the probe over the acoustic window of interest under the direction of an expert sonographer (Figure 1). The participant then held the probe stationary while the sonographer controlled the system remotely to acquire images for analysis.

In addition to teleoperation, the motorized probes and ultrasound system were programed to perform a volume capture (Arbeille et al., 2014b; Arbeille et al., 2018). With this mode activated, the probe moves the transducer (−45 to +45° from vertical) to progressively scan the volume under the probe. The recorded video of the scan is then processed to make a 3D reconstruction of the scanned area that can be later used to perform measurement of difficult to image structures or as a method for determining structure volume.

**FIGURE 2:**
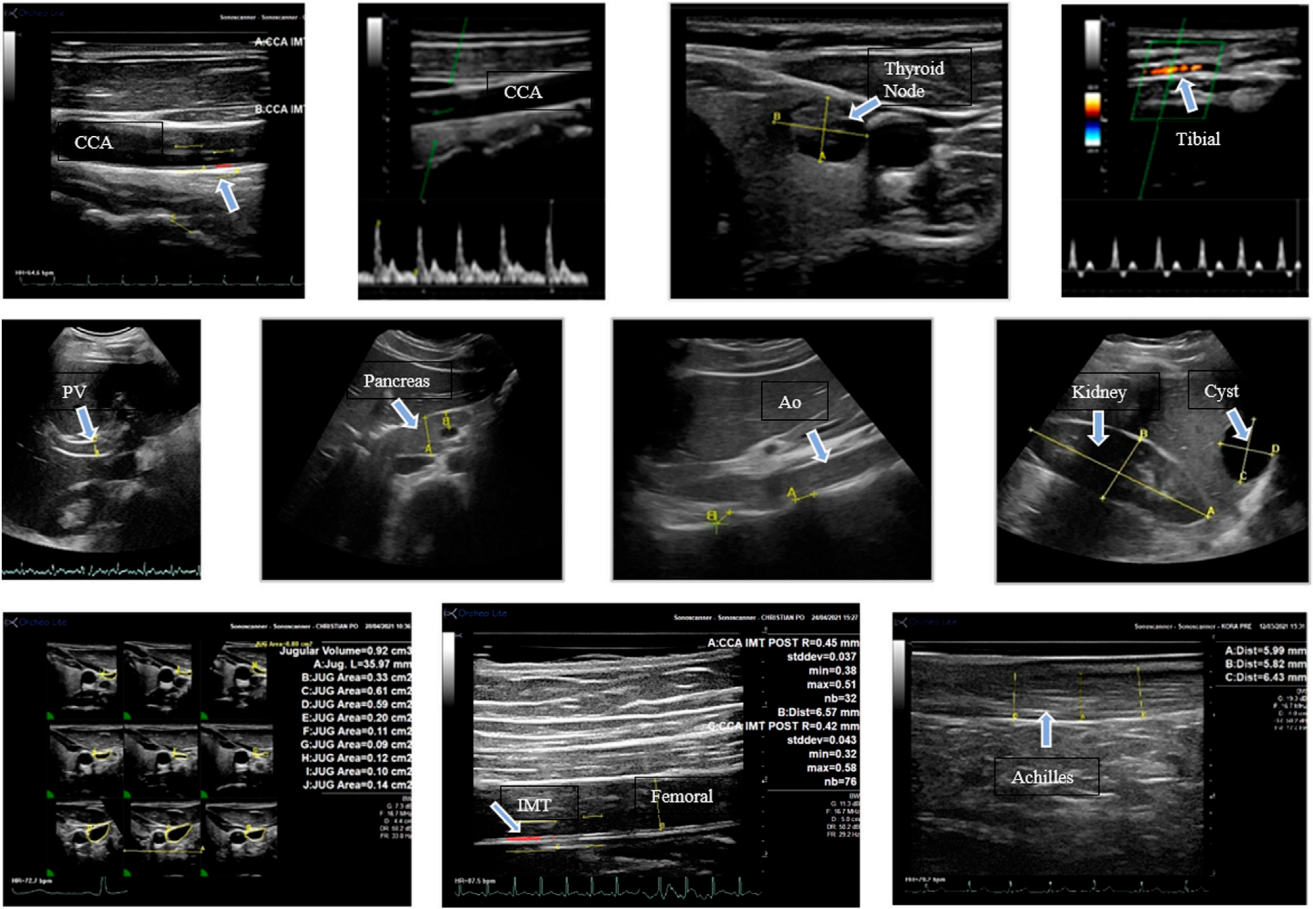
Images with arrows indicating various structures assessed in this study. The images are described from left to right. Top row: longitudinal view of the common carotid artery; (CC) carotid Doppler velocity; thyroid with a nodule; tibial artery Doppler velocity. Middle row: portal vein (A); head of the pancreas; abdominal aorta (Ao) and lumbar vertebral distance; kidney (A-B) and liver cyst (C-D). Bottom row: jugular vein volume measurements from the 3D reconstruction, femoral artery IMT, Achilles tendon measurement.

By using eight different acoustic windows, 12 different structures were assessed with 19 measurements being evaluated before and after isolation (Figure 2). The structures included the common carotid artery (CCA), femoral artery (FA), tibial artery (TA), jugular vein (JV), portal vein (PV), abdominal aorta (Ao), bile duct (BD), kidney, pancreas, cervical vertebrae (CVert), lumbar vertebrae (LVert), and Achilles tendon. For the CCA, FA, and TA Doppler measures were performed for the determination of blood flow responses. In addition to measurements, all organs and vessels assessed were examined qualitatively for structural abnormalities.

### Image evaluation

A longitudinal view of the CCA was obtained for measures of vessel intima media thickness (IMT), systolic and diastolic diameter, peak systolic and end diastolic Doppler velocity, and the radiofrequency (RF) processing (Arbeille et al., 1995). An index of CCA distensibility (DI) was calculated by determining the change in vessel diameter over a cardiac cycle as a percentage of the diastolic diameter. Mean vessel diameter was calculated as the sum of one-third systolic diameter and two-thirds diastolic diameter. Mean blood velocity was also determined using this method and combined with mean diameter for an indication of CCA blood flow. As blood velocity in the CCA is positive throughout the cardiac cycle, the Doppler resistance index was calculated as RI = (systolic velocity—diastolic velocity)/systolic velocity. RF processing (Arbeille et al., 2021a) was conducted along six lines crossing the CCA to provide measures of the index of reflectivity for the anterior wall of the CCA (AW), anterior intima (A-Int), vessel lumen, posterior intima (P-Int), and the posterior wall of the CCA (PW). Finally, while imaging the neck for the CCA measurements, volume capture was performed which allowed for the measurement of JV volume and C Vert distance.

Similar to the CCA, a long axis view of the FA was obtained for measures of vessel diameter, IMT, and peak systolic and diastolic Doppler velocity. As the velocity profile in the FA is indicative of high resistance (biphasic flow component) the high resistance index was calculated as HRI = D/S (Léger et al., 1988) with S systolic peak and D the negative diastolic component. This index was also used for the calculation of resistance from the TA blood velocity trace. FA blood flow was calculated from vessel cross-sectional area and peak systolic velocity.

Abdominal imaging allowed for measurements of PV, Ao, and BD diameter. Kidney area, the head of the pancreas, LVert were also assessed. Finally, the thickness of the Achilles tendon was also determined by imaging the lower leg.

### Fitness assessment

Before entering the cave, on the day of exit and 2 months after leaving the experiment, the physical condition parameters were assessed:

Body composition was determined before and after isolation using bioelectrical impedance (SECA Body Scan MBCA 520) and skinfold measurements (Harpenden clamp) taken at the subscapular, biceps, triceps, and supra-iliac locations.

Maximum aerobic speed (MAS) was determined using the Luc-Léger, 20 m shuttle test (Léger et al., 1988) with heart rate measured continuously throughout the test (Team Polar). Endurance and anaerobic lactic metabolic power was evaluated using the RAST (Zagatto et al., 2008).

### Statistical analysis

All data reported represent mean ± standard deviation. Differences between measures before and after isolation were assessed using paired t-tests (SigmaPlot 12.5, Systat Software Inc., San Jose, CA). Responses were also assessed for a potential effect of sex using a two-way repeated measures analysis of variance. For all tests, significance was set a *p* < 0.05.

## Results

### Body composition

The majority of body composition measurement were not changed with isolation (*p* > 0.05; Body Mass (pre: 66.12 kg, post: 67.14 kg); BMI (pre: 22.37, post: 22.47); Lean Body Mass (pre: 52.15%, post: 51.80%); Skeletal Muscle Mass (pre: 25.37%, post: 24.67%); Body Water (pre: 38.22%, post: 38. 13%); Abdominal Perimeter (pre: 79 cm, post: 80 cm); Subscapular Fold (pre: 13.38 mm, post: 13.92 mm); Biceps Fold (pre: 6.01 mm, post: 5.69 mm); Triceps Fold (pre: 11.93 mm, post: 11.67 mm)). There was an increase in percent body fat (pre: 13.01%, post: 15.16%; *p* < 0.05) and a reduction in supra-iliac skin fold thickness (pre: 12.31 mm, post: 10.08 mm; *p* < 0.05).

### Fitness

Maximum aerobic speed (MAS) decreased slightly with isolation (pre: 11.2 ± 1.0 km/h, post: 10.8 ± 0.8 km/h; *p* < 0.05) as well as test duration (pre: 475 ± 120 s, post: 415 ± 100 s). Power and anaerobic metabolic capacity did not change between pre (average of 6 sprints: 5.4 ± 0.6 s) and post (5.4 ± 0.5 s) periods (*p* > 0.05). Maximum heart rate was also not different between pre (182 ± 8 beats/min) and post (186 ± 8 beats/min) isolation (*p* > 0.05).

### Ultrasound image content and measures

Ultrasound images (Figure 2) were successfully obtained for the majority of participants pre and post isolation (Table 1).

**TABLE 1:** Ultrasound measurements before and after 40 days of isolation.

Structure	Measure	Pre-isolation	Post-isolation
Common carotid artery	IMT (mm)	0.47 ± 0.07	0.49 ± 0.08
	(n = 14)		
	Diameter (cm)	0.63 ± 0.07	0.61 ± 0.07
	(n = 12)		
	Blood flow (mL/min)	363 ± 72	364 ± 82
	(n = 12)		
	Distensibility index (n = 12)	0.13 ± 0.03	0.12 ± 0.03
	Resistance index (n = 14)	0.80 ± 0.06	0.81 ± 0.04
	Index of reflectivity (n = 12)		
Femoral artery	IMT (mm)	0.44 ± 0.05	0.44 ± 0.05
	(n = 10)		
	Diameter (cm)	0.63 ± 0.05	0.63 ± 0.05
	(n = 14)		
	Blood flow (mL/min)	103 ± 19	106 ± 27
	(n = 14)		
	Resistance index (n = 14)	0.43 ± 0.08	0.41 ± 0.10
Jugular vein	Volume (cm^3^)	0.50 ± 0.37	0.47 ± 0.22
	(n = 13)		
Portal vein	Diameter (mm)	11.5 ± 1.6	11.8 ± 1.7
	(n = 14)		
Abdominal aorta	Diameter (mm)	15.5 ± 1.6	15.3 ± 1.7
	(n = 13)		
Bile duct	Diameter (mm)	2.6 ± 0.9	2.6 ± 0.8
	(n = 12)		
Kidney	Area (cm^2^)	41.5 ± 5.1	40.4 ± 4.5
	(n = 13)		
Pancreas	Length (mm)	28.7 ± 3.2	27.9 ± 3.4
	(n = 13)		
Tibial artery	Resistance index (n = 14)	0.30 ± 0.08	0.29 ± 0.10
Vertebrae	Cervical distance (mm)	3.8 ± 0.6	4.2 ± 0.5
	(n = 11)		
	Lumbar distance (mm)	8.6 ± 2.0	7.5 ± 2.4
	(n = 10)		
Achilles tendon	Thickness (mm)	4.8 ± 0.8	4.6 ± 0.8
	(n = 11)		

Values in the table mean ± SD, of the measure before (Pre) and after (Post) 40 days of isolation on the 14 subjects. No significant differences were found between pre and post isolation.

For some participants, poor echogenicity prevented measurements from being completed. The number of participants included in each assessment is indicated for each result. Qualitative assessment of each of the structures investigated found no abnormalities post isolation. Three abnormal structures were identified during pre testing (two thyroid nodes and one liver cyst) but were not different post 40 days in the cavern.

**FIGURE 3:**
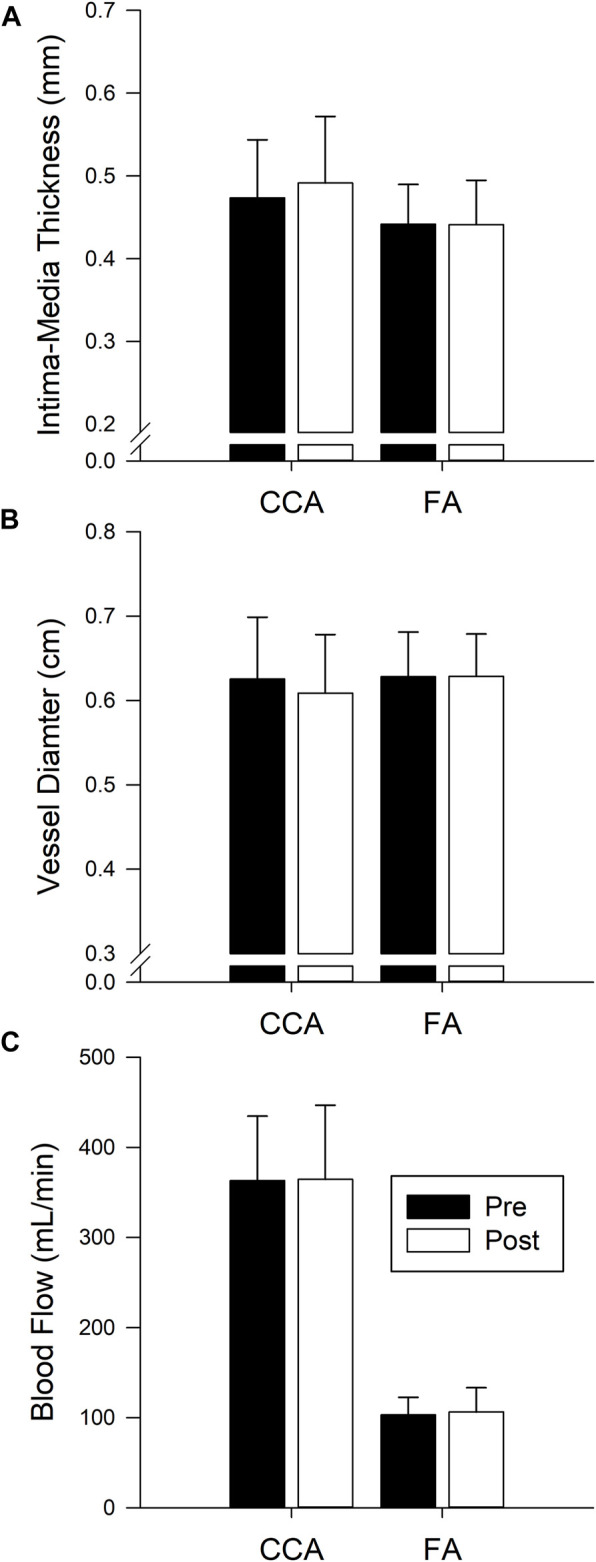
Bars represent the mean ± SD of intima-media thickness **(A)** CCA n = 14, FA n = 10), diameter **(B)** CCA n = 12, FA n = 14), and blood flow **(C)** CCA n = 12, FA n = 14) for the common carotid artery (CCA) and femoral artery (FA) before (Pre, black bars) and after (Post, white bars) 40 days of isolation. No significant difference pre post.

Measures of CCA and FA IMT, diameter, and blood flow are presented in Figure 3. CCA IMT, diameter, and blood flow were not different pre to post isolation (*p* = 0.405, *p* = 0.499, and *p* = 0.976 respectively). Similarly, IMT, diameter and flow of the FA were not changed (*p* = 0.94, *p* = 0.962, and *p* = 0.725 respectively). Additionally, there was no change in the Carotid distensibility index (*p* = 0.614).

**FIGURE 4:**
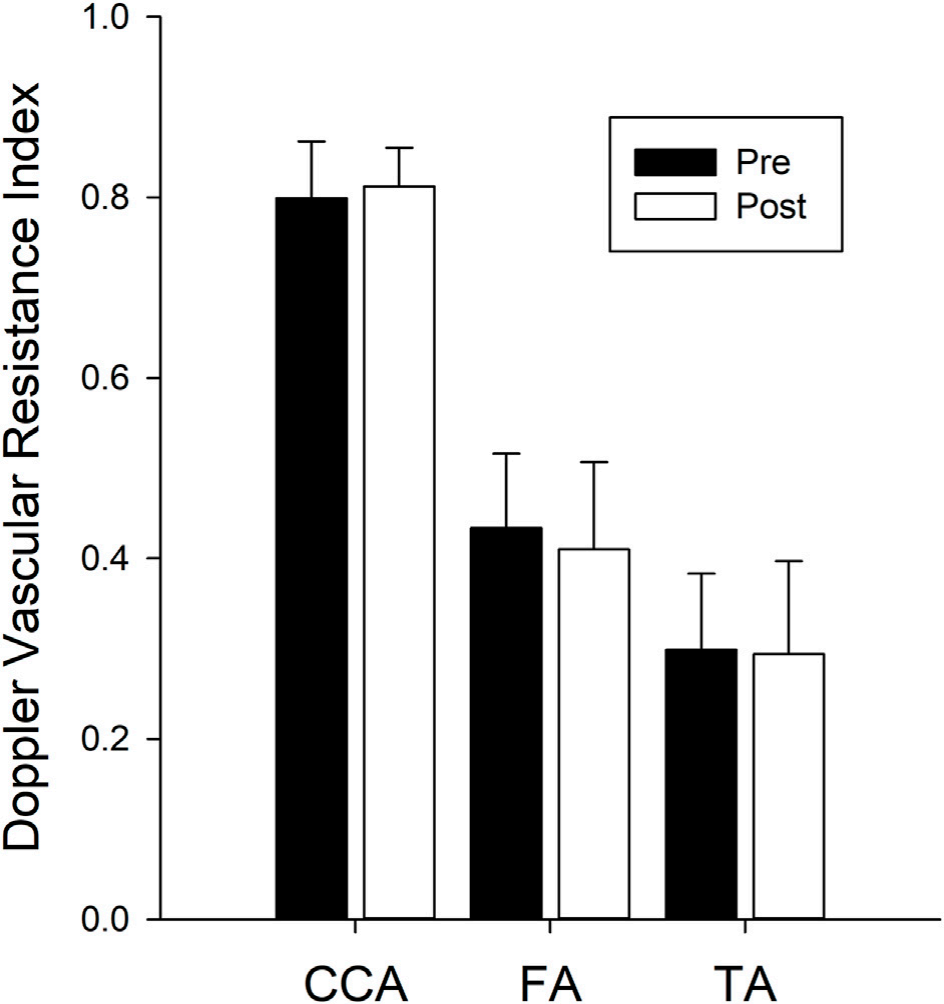
Bars represent the mean ± SD for the Doppler vascular resistance indices (n = 14 for all three vessels) calculated for the common carotid artery (CCA), femoral artery (FA), and tibial artery (TA). There were no differences between values calculated pre isolation (black bars) and post isolation (white bars).

**FIGURE 5:**
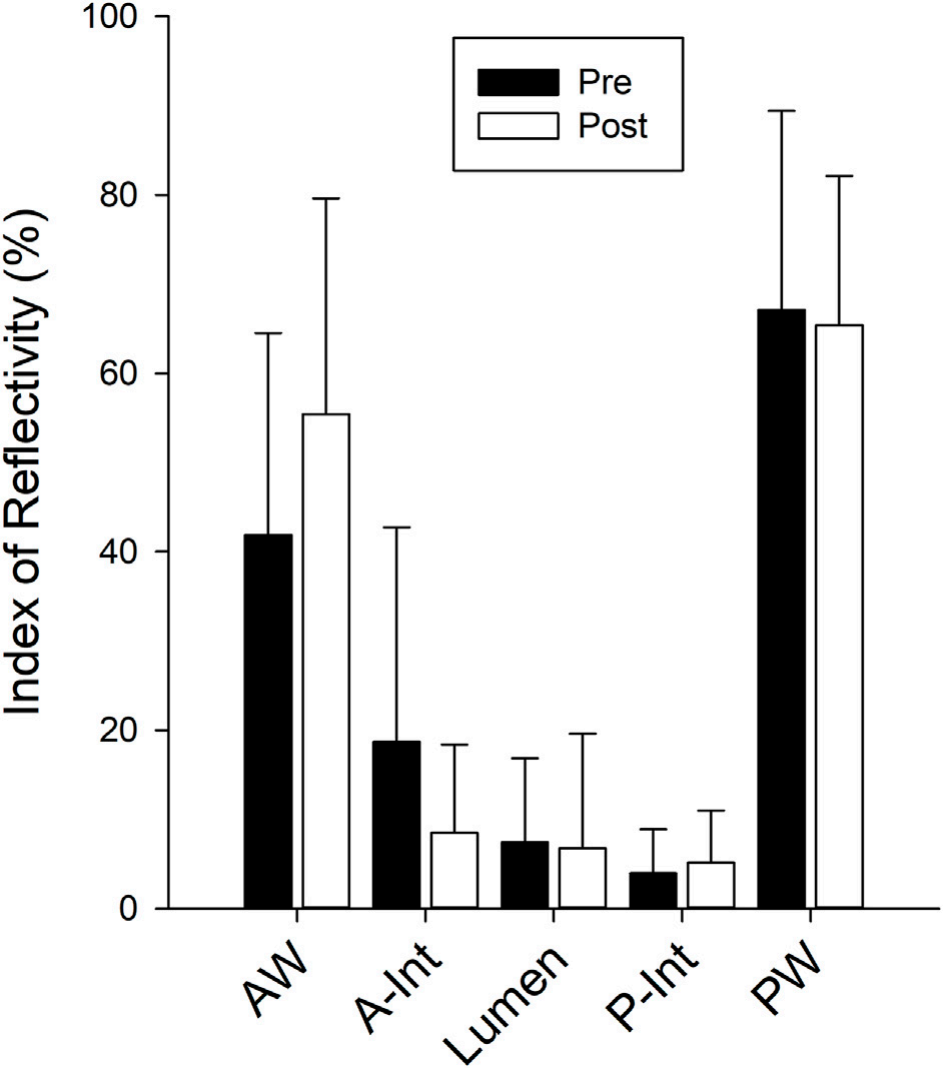
Bars represent the mean ± SD for the Carotid index of reflectivity calculated from the recorded radiofrequency signal for the anterior wall (AW), anterior intima (A-Int), vessel lumen, posterior intima (P-Int), and posterior wall (PW) of the common carotid artery (n = 12 for all measures). There were no differences between values calculated pre isolation (black bars) and post isolation (white bars).

The Doppler resistance indices (Figure 4) were not changed with isolation for the CCA (*p* = 0.238), FA (*p* = 0.345), or TA (*p* = 0.775). The index of reflectivity was also not altered with isolation (Figure 5) for AW (*p* = 0.106), A-Int (*p* = 0.224), vessel lumen (*p* = 0.887), P-Int (*p* = 0.632), or PW (*p* = 0.644). Measures of the other structures investigated (Table 1) also found no effect of isolation. No effects of sex were found for any of the variables assessed.

## Discussion

The Deep Time experiment was designed to simulate the isolation and confinement anticipated to be experienced by astronauts during space exploration missions and evaluate if the particular environmental conditions (cavern volume, number of subjects, high sustained physical activity) may influence the adaptation. Therefore, the purpose of the current study was to determine the effects of 40 days of isolation on blood vessel and organ structure assessed using ultrasound. Contrary to the findings of altered arterial wall structure reported after 30 days during the confinement of spaceflight (Hughson et al., 2016; Arbeille et al., 2017) and after 60–90 days during simulated space exploration on Earth during the Mars 500 study (Arbeille et al., 2014a) and CELSS confinement study (Yuan et al., 2019), no changes were found in any participant for common carotid artery IMT, distensibility, and reflectivity as well as for femoral artery intima-media thickness. Nevertheless we cannot exclude that 40 days isolation in the present condition was not sufficient to produce the changes seen on vascular structures after 60–90 days of confinement on Earth or with ISS spaceflight. Additionally, the isolation protocol utilized in the present study did not results in alteration to other organs and vascular structures that have yet to be assessed during long duration confinement and spaceflight. Lastly there was no differences between women and men results.

As the Deep Time experiment was designed as a simulated space exploration mission, the current study utilized teleoperated ultrasound to mimic systems currently available on the ISS (Arbeille et al., 2016; Arbeille et al., 2018). Using the teleoperated system and volume capture feature, the expert sonographer away from the subject was able to obtain long and short axis views of each structure of interest and record 2D and 3D video for later assessment. This greatly reduced the time required for capturing an extensive investigation on a participant holding the probe who was unfamiliar with ultrasound operation to within 50 min. Additionally, images collected via tele echography using this method were equivalent in quality to those collected by a trained sonographer as has previously been demonstrated in remote medical centres and on the ISS (Arbeille et al., 2016; Arbeille et al., 2018). Similar ultrasound protocol (Tele-echography of the same organs) will be run onboard ISS during long duration spaceflight as part of the CIPHER program (name: Routine Ultrasound).

Exposure to real and simulated microgravity results in several vascular adaptations. At the level of the head and neck, studies have found increased carotid IMT and stiffness (Arbeille et al., 2014a; Hughson et al., 2016; Arbeille et al., 2017; Yuan et al., 2019) as well as increased jugular vein volume (Arbeille et al., 2015; Marshall-Goebel et al., 2019; Arbeille et al., 2021b) and increased cerebral venous velocity (Arbeille et al., 2021b) potentially contributing to increased intracranial pressure and observed changes in vision (Mader et al., 2011; Laurie et al., 2020). Portal vein volume increases (Arbeille et al., 2015) indicating splanchnic blood pooling with potential implications for abdominal organs. Spinal changes may also occur with spaceflight as astronauts frequently report back pain and have a greater risk of intervertebral disc herniation after spaceflight (Belavy et al., 2016). The current study aimed to investigate multiple organs, blood vessels, and structures to determine if similar changes develop from the stress of long-term isolation on Earth.

Observed changes in blood vessel structure and function with spaceflight have been thought to be the result of headwards fluid shifts and cardiovascular unloading that occurs with microgravity exposure. However, two previous space exploration simulation studies in 1 G (Mars 500 and CELSS Arbeille et al., 2014b; Yuan et al., 2019)) which only involved confinement, found increases in common carotid and femoral artery IMT suggesting that the stress of confinement and the lack of physical activity may greatly contribute to physiological adaptations. Contrary to the hypothesis, the results of the current study indicated no changes in carotid or femoral artery IMT or wall properties. Interestingly, the carotid index of reflectivity (Arbeille et al., 2021a), which increased after 6 months of spaceflight and after only 4 days in dry immersion, remained unchanged in the present study. The absence of any change in the index of reflectivity confirmed a lack of change in carotid artery wall structure and aligned with the lack of change in IMT and distensibility.

Several factors associated with the current study could explain the lack of observed adaptations in contrast to previous Earth-based isolation studies. The duration of the current study, 40 days, was shorter than previous confinement studies but vascular changes in the CELSS study were found after 60 days of confinement (Yuan et al., 2019).

Additional differences between the current study and previous studies are with respect to the isolation environment and study participants. The cavern used for the current study was much larger than the habitats use in Mars 500, CELSS, or the environment on the ISS. Additionally, there were more people and both sexes involved in the current study. Together, these factors may reduce the stress of the isolation environment as participants were able to socialize with a greater number of individuals and had space to isolate if desired from the other study participants.

The Deep Time experiment also involved a greater level of physical activity than in any other confinement or spaceflight studies. The sleeping area for participants was located 800 m away from the living space requiring participants to walk this distance at least twice a day. Participants also regularly performed exploration and water recovery missions which required several hours of physical activity to complete each of these missions. While aerobic endurance decreased slightly with confinement (5% drop in VMA), maximal heart rate and anaerobic parameters were not changed with isolation. This suggests that the daily activity performed by participants were sufficient to maintain physical fitness and may have provided a protective effect preventing physiological changes with isolation.

## Conclusion

The current study found no significant physiological effects on the vascular and organ structures assessed with ultrasound after 40 days of isolation within a cavern. In contrast to previous studies which found alterations in vascular structure after 60 days of confinement on Earth, results from the current study suggest that a large living area, multiple companions, and regular and sustained physical activity may reduce the stress of confinement and provide protective effects against negative adaptations to isolation and confinement. However, it is possible that vascular and organ adaptations may occur after longer durations of isolation with potential sex differences in responses. Further work is needed to investigate these factors in preparation for longer duration space exploration missions to the moon and Mars.

## Data Availability

The original contributions presented in the study are included in the article/supplementary material, further inquiries can be directed to the corresponding author.
